# Maintenance vs. Manipulation in Auditory Verbal Working Memory in the Elderly: New Insights Based on Temporal Dynamics of Information Processing in the Millisecond Time Range

**DOI:** 10.3389/fnagi.2020.00194

**Published:** 2020-07-23

**Authors:** Katarzyna Jablonska, Magdalena Piotrowska, Hanna Bednarek, Aneta Szymaszek, Artur Marchewka, Marek Wypych, Elzbieta Szelag

**Affiliations:** ^1^Faculty of Psychology, SWPS University of Social Sciences and Humanities, Warsaw, Poland; ^2^Laboratory of Neuropsychology, Nencki Institute of Experimental Biology of the Polish Academy of Sciences, Warsaw, Poland; ^3^Laboratory of Brain Imaging, Nencki Institute of Experimental Biology of the Polish Academy of Sciences, Warsaw, Poland

**Keywords:** working memory, temporal information processing, auditory *n*-back task, fMRI, maintenance and manipulation processes

## Abstract

Working memory (WM) is a limited-capacity cognitive system that allows the storage and use of a limited amount of information for a short period of time. Two WM processes can be distinguished: maintenance (i.e., storing, monitoring, and matching information) and manipulation (i.e., reordering and updating information). A number of studies have reported an age-related decline in WM, but the mechanisms underlying this deterioration need to be investigated. Previous research, including studies conducted in our laboratory, revealed that age-related cognitive deficits are related to decreased millisecond timing, i.e., the ability to perceive and organize incoming events in time. The aim of the current study was: (1) to identify in the elderly the brain network involved in the maintenance and manipulation WM processes; and (2) to use an fMRI task to investigate the relation between the brain activity associated with these two processes and the efficiency of temporal information processing (TIP) on a millisecond level reflected by psychophysical indices. Subjects were 41 normal healthy elderly people aged from 62 to 78 years. They performed: (1) an auditory verbal *n*-back task for assessing WM efficiency in an MRI scanner; and (2) a psychophysical auditory temporal-order judgment (TOJ) task for assessing temporal resolution in the millisecond domain outside the scanner. The *n-back* task comprised three conditions (0-, 1-, and 2-back), which allowed maintenance (1- vs. 0-back comparisons) and manipulation (2- vs. 1-back comparisons) processes to be distinguished. Results revealed the involvement of a similar brain network in the elderly to that found in previous studies. However, during maintenance processes, we found relatively limited and focused activations, which were significantly extended during manipulation. A novel result of our study, never reported before, is an indication of significant moderate correlations between the efficiency of WM and TIP. These correlations were found only for manipulation but not for maintenance. Our results confirmed the hypothesis that manipulation in the elderly is a dynamic process requiring skilled millisecond timing with high temporal resolution. We conclude that millisecond timing contributes to WM manipulation in the elderly, but not to maintenance.

## Introduction

Working memory (WM) is involved in almost every cognitive task and plays a crucial role in complex cognition in humans. It allows one to successfully keep and manipulate information in mind over a short period of time (Baddeley, 2000; Cowan, 2008). Because of the important role of such moment-to-moment processing in mental activity, impairments in WM function can be a major barrier to independent living. One of the possible causes of a decline in WM efficiency may be the neurodegenerative processes associated with normal aging, known also as “healthy aging” (for a summary see Fakhri et al., 2013; Nyberg et al., 2014; Eriksson et al., 2015). Many people with advancing age report problems in processing information in rapidly changing contexts. Evidence indicates that WM consists of two different processes: *maintenance* for temporary storage in a readily accessible state and *manipulation* for the online information processing required for the guidance of subsequent behavior. Accordingly, maintenance has been defined as storage (including rehearsal), monitoring, and matching information in WM, whereas manipulation refers to the reorganization and updating of each memory set (Fletcher and Henson, 2001; Veltman et al., 2003).

As these processes differ in their sensitivity to advancing age, one may observe individual differences in WM. On this matter, Dixon and de Frias (2007) and Lindenberger et al. (2013) reported an increase in individual differences in WM in the elderly in comparison with those observed in young adults presumably reflecting the contribution of general cognitive domains—such as sustained and executive attention or short- and long-term memory—to WM efficiency (Conway et al., 2005; Jaeggi et al., 2010). This leads to the question: why is WM better preserved in some elderly people than in others?

The main objective of the present study is to understand such individual differences in elderly subjects with reference to temporal information processing (TIP). The rationale for this approach comes from a number of literature studies, including studies conducted in our laboratory, which indicate that TIP in the millisecond range sets a frame for our mental activity and determines human behaviors (e.g., Szelag et al., 2011; Bao et al., 2013, 2014; Nowak et al., 2016). Many mental functions, including WM, may be characterized by their specific temporal dynamics in the millisecond domain; hence, patterning in time is considered to be one of the characteristic features of our working brains (Lewandowska et al., 2010; Radua et al., 2014; Nowak et al., 2016). One may assume, therefore, that TIP constitutes a neural basis for mental activity in both normal and pathological conditions, including cognitive declines in healthy aging (e.g., Szymaszek et al., 2009; Teixeira et al., 2013). Accumulated data have also indicated age-related deterioration in TIP (e.g., Fitzgibbons and Gordon-Salant, 1994; Kolodziejczyk and Szelag, 2008; Szymaszek et al., 2009; Kumar and Sangamanatha, 2011; Nowak et al., 2016).

The temporal dynamics of the processes underlying WM are supported by the original Scalar Expectancy Theory (Treisman, 1963; Treisman et al., 1990; Meck, 2005; Matthews and Meck, 2016). These authors assumed that TIP is associated with the operation of three mental stages, i.e., internal clock, memory (WM and reference memory), and decision processes. Thus, individual differences in timing might reflect alterations within these stages. This suggests that cognitive functions cannot be understood without their temporal frame. Despite the fact that WM studies require efficient TIP and vice versa, the interface of timing and WM has been rarely studied in the existing literature (Lewandowska et al., 2010; Radua et al., 2014; Üstün et al., 2017).

Given the importance of TIP for our mental activity, the present study focuses on relationships between WM and TIP in advanced age. Both these functions seem to deteriorate in the elderly. Based on neuroimaging data (see below), these relationships seem to be of great importance for understanding individual differences in WM—in particular, why some people have more efficient WM than others. One might hypothesize that the two WM processes, i.e., maintenance and manipulation, are differentially vulnerable to TIP (see below for more explanation).

### Neuroanatomical Representation of WM and TIP

Recent neuroimaging data have indicated the involvement of several brain areas in WM tasks, stressing that a key role is played by the *prefrontal cortex* (PFC; Ragland et al., 2002; Tisserand and Jolles, 2003; Raz et al., 2005; Fuster, 2009; Fakhri et al., 2013; Lindenberger et al., 2013; D’Esposito and Postle, 2015; Luis et al., 2015). In particular, three regions of the lateral PFC—*ventrolateral* (vlPFC), *dorsolateral* (dlPFC), and *anterior* (aPFC)—are consistently reported to be involved (Fletcher and Henson, 2001). The involvement of more posterior areas has been also postulated, i.e., the *premotor*, *parietal*, *cingulate* and *superior temporal gyrus*, and *supplementary motor area* (SMA). Furthermore, some authors indicated the role of the *basal ganglia* (BG) and *cerebellum*, as well as regions specialized for processing particular modalities (see the meta-analysis of 24 previous studies by Veltman et al., 2003; Owen et al., 2005; D’Esposito and Postle, 2015; Luis et al., 2015; for the recent overview see also Eriksson et al., 2015). Some studies have found that brain activity during WM is vulnerable to attentional load, the type of information to be maintained, as well as perceptual and long-term memory representations. Hence, the neurocognitive architecture of WM results also from the specific complex interactions within a given task (e.g., Jaeggi et al., 2010; Eriksson et al., 2015; summarized in Box 1; Luis et al., 2015).

Another support for the “WM–TIP” relation comes from the neural underpinnings of timing, which indicate the involvement of many brain areas in TIP (for a recent review see Merchant et al., 2013). One approach assumes that the main core timing mechanism interacts with context-dependent areas. This hybrid model suggests a partially distributed timing mechanism, integrated by core structures such as *cortico-thalamo-BG* circuits and areas that are selectively engaged in a given task (Buhusi and Meck, 2005; Coull et al., 2011; Matthews and Meck, 2014). Such diffuse timing representation is in agreement with the earlier report by Rao et al. (2001), who indicated the involvement of nontemporal processes during timing tasks, reflected by activations in the dlPFC (related to WM or comparison functions), posterior parietal cortex, and anterior cingulate cortex (ACC, which controls modulatory attention), as well as the BG providing the subcortical “timekeeper” system. These results are supported by numerous and more recent fMRI studies, which have consistently identified several key timing areas: the right inferior frontal cortices, in particular, the inferior prefrontal cortex, dlPFC, ACC, SMA, and nonfrontal brain regions such as the inferior parietal lobules (IPLs), cerebellum, and BG (Coull and Nobre, 2008; Szelag et al., 2009; Allman and Meck, 2012; Merchant et al., 2013). The functional contribution of these areas to the timing network is still a matter of debate.

The theoretical dissociation of the processes underlying WM is based on neuroimaging data indicating the neuroanatomical segregation of maintenance and manipulation processes. This distinction between the activations obtained for these two processes seems important in the context of studying the role of TIP in WM. We hypothesize that TIP is more important for processing information within WM than for maintenance because of the more dynamic mental process involved in reordering and updating. Therefore, analyses of brain activity during each of these two processes may provide a better understanding of the role of TIP in WM.

### Neuroanatomical Representation of Maintenance and Manipulation Processes

The early approach to this distinction (Smith et al., 1998; Veltman et al., 2003) was based on a comparison of brain activity between the traditional item-recognition tasks (e.g., the Sternberg WM task)—classified as pure storage tasks (involving rehearsal and storage)—and the *n*-back task (characterized more as a manipulation task involving processing and updating). Based on such comparisons, Smith et al. (1998) indicated that the dlPFC is activated whenever a WM task requires processing. In contrast, the traditional item-recognition tasks (which can be considered as storage tasks) did not produce any sign of dlPFC activation.

On the other hand, Veltman et al. (2003) indicated the functional rather than neuroanatomical distinction between maintenance (increased primarily on the Sternberg task) and manipulation (increased on the *n*-back task) processes, given the evidence that both these tasks resulted in a very similar pattern of task-related activations in the bilateral dlPFC, left vlPFC, bilateral parietal cortex, as well as in the cerebellum and SMA.

The other approach to explaining the neuroanatomy of WM processes comes from studies exploring *n*-back tasks. Over the years, these tests have been frequently used as continuous-recognition measures that present stimulus sequences, such as letters, pictures, syllables, etc. (e.g., Miller et al., 2009; Jaeggi et al., 2010). The subjects’ task is to judge for each item in the sequence whether it matches the one presented *n*-items before. Participants must maintain and update a dynamic rehearsal set while responding to each item. Behavioral data using the *n*-back task have demonstrated that the reason for the “2-back” condition being more difficult than the “1-back” is that these conditions involve different processes associated with WM. Specifically, the “1-back” task is based more on maintenance, whereas “2-back” uses manipulation resources.

Recent neuroimaging studies have indicated the involvement of several brain areas in these two conditions (D’Esposito et al., 1999; Rypma and D’Esposito, 1999; Tsukiura et al., 2001; Ragland et al., 2002). Using the *n*-back task, a few contrasts may be designed to reveal activation changes reflecting maintenance vs. manipulation demands (Ragland et al., 2002). Specifically, the “1- vs. 0-back” contrast reveals changes related to WM maintenance (controlling for perceptual and motor components) while minimizing the role of the central executive system and updating (manipulation) resources. On the other hand, the “2- vs. 0-back” contrast shows activation changes reflecting the addition of central executive components (monitoring and manipulation) to the online maintenance demands. On the contrary, the “2- vs. 1-back” contrast diminishes the effect of maintenance demands (involved in both 1- and 2-back tasks) and allows the gauging of WM manipulation resources, given the difficulty of the task. The application of these three contrasts allows the identification of the involvement of specific brain areas in different WM processes.

Both the existing theoretical models and the results of neuroimaging studies have compared the specific functions, i.e., maintenance of sensory information vs. manipulation for which the central executive components are required (Petrides, 1994; D’Esposito et al., 1998). Whereas the former WM processes involve the vlPFC (BA 44, 45, and 47), the latter ones activate the dlPFC (BA 9 and 46). This functional dissociation, however, appears rather to be relative than absolute (Ragland et al., 2002).

### Experimental Aim

The present study addresses the question of whether individual differences in brain activity in normal healthy elderly subjects during maintenance and manipulation of information in WM are related to the efficiency of TIP evidenced in psychophysical indices. We hypothesize that WM and millisecond timing are interrelated and may share a similar neuroanatomical basis. However, fluctuations in cognitive efforts to perform more difficult WM tasks may influence this relationship. Our study has the following two aims:

(1) To identify in elderly subjects the brain structures engaged in maintenance (storage, monitoring, and matching) of presented auditory verbal material, as well as in the manipulation (reorganization and updating) of such material, using the standard *n*-back auditory task.
(2) To investigate the relationship between the efficiency of millisecond TIP and WM by examining both behavioral indices and brain activity in the two WM processes. Taking into account that maintenance is reflected more in storage and manipulation in dynamic processing, we hypothesize that TIP is related mostly to manipulation but not to maintenance. The relationships between TIP and manipulation could provide a new insight into individual differences in WM in the elderly.

To avoid any cross-modal interference that might obscure the observed relationships, both the TIP and WM measurements applied here employ the perception of auditory stimuli.

## Materials and Methods

### Participants

Forty-one normal healthy elderly adults (37 women and four men), aged from 62 to 78 years (*M* = 67.1, *SD* = 3.7) took part in the study. They were recruited through local advertisements at various community centers, senior clubs, and Universities of the Third Age in the Warsaw area. All participants had between 11 and 18 years of education and were right-handed Polish native speakers. They reported no neurological or psychiatric disorders, head injuries, systemic diseases, or the use of medications affecting the central nervous system. The above-mentioned inclusion and exclusion criteria were verified in an interview with each subject individually. Furthermore, hearing level was screened using pure-tone audiometry (Audiometer MA33, MAICO; ANSI 2004). The tested frequencies were selected to encompass the frequency spectrum of the presented auditory stimuli, which included 250, 500, 750, 1,000, 1,500, 2,000, and 3,000 Hz. All subjects had normal hearing level, assessed with pure-tone average (PTA), i.e., ≤25 dB HL at 500, 1,000, and 2,000 Hz (Carhart, 1971; Kung and Willcox, 2007).

The Mini-Mental State Examination (MMSE; Folstein et al., 2001) and the Geriatric Depression Scale (GDS; Sheikh and Yesavage, 1986) were used to screen for dementia and depression. A score of 27 or more points on the MMSE (*M* = 28.93, *SD* = 1.01) and a score of 5 or fewer points on the GDS (*M* = 2.61, *SD* = 1.75) were inclusion criteria. The study was approved by the Ethical Commission at the University of Social Sciences and Humanities (permission no 1/2017, registered as 2 /I/ 16–17) and was in line with the Declaration of Helsinki. All participants signed a written informed consent form prior to the study.

### Experimental Paradigms

Each participant completed two experimental paradigms performed in separate sessions. These paradigms comprised: (1) an auditory *n*-back fMRI task; and (2) a psychophysical auditory temporal-order judgment (TOJ) task.

#### Auditory *n*-Back fMRI Task

**Figure 1:**
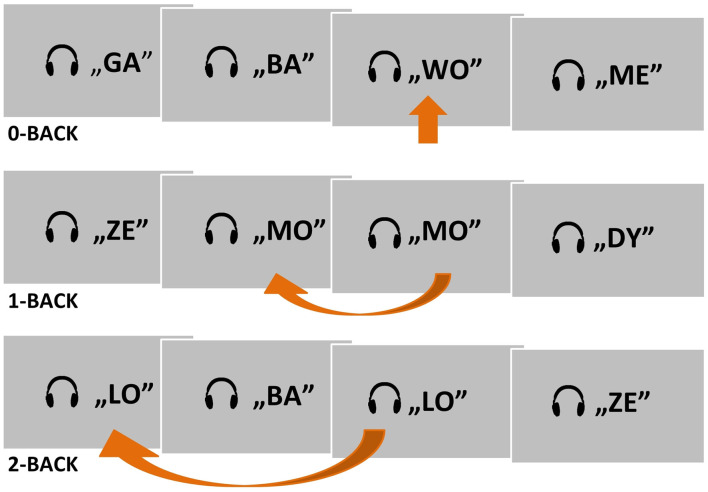
A schema of the two experimental conditions (1- and 2-back) and one control condition (0-back) used in the auditory *n*-back fMRI task. Target situations are indicated by arrows.

A simple block design with two experimental *n*-back conditions (1- and 2-back) and one control condition (0-back) was applied (Figure 1). The auditory stimuli were 30 consonant–vowel syllables lasting 300 ms each. The successive syllables were separated by 1,700-ms silent intervals. Each syllable was built from one of six consonants (B, D, G, L, M, Z), followed by one of five vowels (A, E, O, U, Y). An additional syllable (WO) was created for the control condition (0-back). The strings of syllables were delivered binaurally through MRI compatible headphones, using Presentation^®^ software (Version 18.0, Neurobehavioral Systems, Inc., Berkeley, CA, USA^1^).

The subjects were asked to listen to presented strings of syllables and to press a button with their right thumb in response to a target syllable (Figure 1). Depending on the condition, the target was the predefined syllable WO (0-back), a syllable matching the previous one (1-back), or a syllable matching the one before the previous syllable (2-back). Subjects were instructed to suppress their reactions to nontarget syllables. A written instruction for the current task was displayed permanently on the screen as a reminder.

The study comprised three sessions. Each session consisted of six blocks: four experimental (1- and 2-back conditions repeated twice) and two control blocks with 0-back condition. During each session, the blocks were presented in pseudo-random order. The example order of blocks in session 1 was as follows: 0-, 1-, 2-, 1-, 0-, and 2-back. Each block lasted 60 s and consisted of 30 trials (five targets and 25 nontargets). Each trial comprised one syllable presentation. The whole study comprised 540 trials.

Measures of behavioral performance on the 0-, 1-, and 2-back conditions were accuracy of performance (reflected in the percentage of correct responses to target syllables in each condition) and reaction time (RT), measured from the onset of the presented syllable to the onset of the subject’s button press for the correct target identification. The percentage of correct responses and the mean RTs from each condition were submitted to data analysis.

To familiarize subjects with the task proper, each participant took part individually in a training session performed outside the scanner. In this session, subjects were presented with 0-, 1-, and 2-back conditions and practiced the detection of a target syllable by pressing a button. After completion of each condition, feedback on correctness achieved was provided. The introductory session was continued until a criterion of 75% correct responses was achieved. Then, the fMRI experiment started. No feedback on performance was provided during the fMRI session.

The MRI procedure was conducted in the Laboratory of Brain Imaging at the Nencki Institute of Experimental Biology using a 3-T Siemens MAGNETOM Trio system (Siemens Medical Solutions). Prior to the task, participants were asked to complete an MRI safety questionnaire.

#### Psychophysical Auditory Temporal-Order Judgment Task

To study the efficiency of TIP in auditory perception, we applied the TOJ task using the psychophysical procedure described in our previous studies (e.g., Szymaszek et al., 2009; Szelag et al., 2018). Some of the results presented here have been previously published in a study that focused on sequencing abilities in different TOJ tasks (Szelag et al., 2018).

Participants were presented with pairs of 10-ms sinusoidal tones with a rise-and-fall time of 1 ms. The stimuli were generated with a Realtek ALC3246 sound controller and Waves MaxxAudio Pro software. They were delivered binaurally at a comfortable listening level through Philips SHP8500 headphones. Each pair consisted of two tones (a low 400-Hz tone and a high 3,000-Hz tone) separated by a short interstimulus interval (ISI)—the time gap between the offset of the first stimulus and the onset of the second stimulus. Two tones within each pair were adjusted to equal loudness on the basis of isophones. A warning signal was delivered 1 s prior to each pair of stimuli in order to focus the participants’ attention on the task. They were asked to report verbally the order of two sounds within the presented pair, i.e., to judge whether the order of tones was high–low or low–high.

The TOJ task consisted of two parts. Part 1 comprised 20 trials (presented tone pairs). The two tones in each pair were separated by fixed ISIs, changing in steps of 27 ms in the range from 240 to 1 ms, in a decreasing (*n* = 10) and then increasing (*n* = 10) order. On the basis of these predefined 20 trials, the ISI value of the first trial in part 2 of the TOJ task was computed according to an algorithm based on maximum likelihood estimation (Treutwein, 1997). Part 2 consisted of 50 trials. The ISI in each trial was adjusted according to an adaptive algorithm, depending on the correctness achieved in the previous response (see Szelag et al., 2018 for a more detailed description).

On the basis of 70 completed trials (20 trials in part 1 and 50 trials in part 2) an auditory temporal-order threshold (ATOT) was calculated (in milliseconds) for each subject (see Szelag et al., 2018). The ATOT reflected the index of TIP efficiency and was defined as the shortest time gap between two paired tones presented in rapid succession at which a subject could identify their temporal order (i.e., their before–after relation) with at least 75% correctness (Szelag et al., 2011, 2014, 2015a,b; Bao et al., 2013, 2014).

In order to familiarize participants with the TOJ task, prior to the task proper, they were instructed by the experimenter and performed a few practice trials. After each response, feedback on correctness was given. During the proper task, no feedback on correctness was given.

The TOJ task was conducted in a soundproof room in the Laboratory of Neuropsychology at the Nencki Institute of Experimental Biology.

### fMRI Data Acquisition and Preprocessing

MRI data were collected on a Siemens 3-T Trio MRI scanner using a 12-channel coil. High-resolution (1 × 1 × 1 mm voxels) T1-weighted anatomical images were acquired with the following parameters: repetition time (TR) = 2,530 ms, echo time (TE) = 3.32 ms, flip angle = 7°, 176 1-mm thick slices, field of view (FOV) = 256 × 256 mm. Functional images (3 × 3 × 3 mm voxels) were acquired using an echo planar imaging pulse sequence with the following parameters: TR = 2,000 ms, TE = 30 ms, flip angle = 90°, 36 interleaved slices with 0.5-mm gap, FOV = 216 × 216 mm. Additionally, a field map was acquired to allow for field inhomogeneity distortion correction, with TE1/TE2/TR = 4.5/6.96/600.

MRI data were preprocessed and analyzed in SPM12^2^. Data from each subject underwent the same preprocessing steps (motion correction and unwarping, coregistration of the T1 image to mean EPI image, and segmentation of the T1 image to different tissues). The fMRI data were normalized to MNI space with 2 × 2 × 2-mm voxels using a standard SPM12 algorithm and smoothed with a 6-mm Gaussian kernel.

## Statistical Analyses

### *n*-Back Behavioral Data and Psychophysical TOJ Indices

The behavioral data from the *n*-back task (accuracy of performance and mean RT), as well as psychophysical TOJ indices (reflected in ATOT values), were analyzed using IBM^®^ SPSS^®^ Statistics 25. As the distribution of data deviated from the Gaussian (according to the Shapiro–Wilk test), nonparametric statistical methods were used. First, to test whether there were differences in the behavioral measures obtained in 0-, 1-, and 2-back conditions, the Friedman test followed by the Wilcoxon signed-rank test were applied. Next, to investigate correlations between the efficiency of WM and TIP, two-tailed Spearman’s rank correlations were used. To control for the effect of subjects’ age on these relationships, partial correlations were performed.

### *n*-Back fMRI Data Analysis

There were two steps in the fMRI data analysis. First, we identified brain areas engaged in maintenance (storage) or manipulation (updating) of the presented material. Second, we investigated the correlations between brain activity during the WM task and psychophysical indices of TIP efficiency.

#### Identification of Brain Structures Engaged in WM Maintenance or Manipulation Processes

The fMRI data analysis was performed with SPM12 software, within the general linear model (GLM) framework. At a single-subject level, design matrices with 0-, 1- and 2-back conditions and additional head movement regressors and regressors of motion affected volumes (acquired with the ART toolbox^3^) were estimated. Images were regarded as artifactual when mean image intensity *z*-threshold was above 9, movement threshold was above 2 mm, or rotation threshold was above 0.02 rad. As a result, on average, 5.05 images per person were identified as outliers (between 0 and 57; however, in no case did it exceed 10% of all images, thus no subject was excluded due to excessive motion). Subsequently, a group-level full factorial model with one within-subjects factor—“task” (0-, 1-, 2-back)—was computed. For whole brain analyses, a gray matter mask of 0.20 probability was applied. Familywise error (FWE) *p* < 0.05 threshold at voxel level was set to correct for multiple comparisons. To indicate brain structures engaged in maintenance and manipulation processes, three *t*-contrasts were computed within the model: 1- vs. 0-back, 2- vs. 1-back, and 2- vs. 0-back (see “Introduction” section for a description of these contrasts). The xjView toolbox^4^ was used to generate tables of activated regions. All activation peaks were labeled using the Talairach Atlas labels (Lancaster et al., 2000). Structural images of each subject were assessed by a radiologist to exclude brain pathology.

#### Correlations Between Brain Activity in WM and Psychophysical Indices of TIP Efficiency

We created regions of interest (ROIs) on the basis of two *t*-contrasts from the group-level model: 1- vs. 0-back and 2- vs. 1-back based on clusters surviving the FWE correction (Poldrack, 2007). The 2- vs. 0-back contrast was not considered in correlation analysis because of the engagement of both WM processes (maintenance and manipulation). In total, 26 functional ROIs were created. Eight ROIs (Table 1) were built on the basis of the 1- vs. 0-back comparison (reflecting maintenance in WM) and 18 on the basis of the 2- vs. 1-back comparison (reflecting manipulation in WM). Additionally, a sphere ROI was built in the visual cortex in the occipital lobe (20 mm in diameter; the MNI coordinates of the center were *x*, 10; *y*, −80; *z*, 20) as an ROI for statistical control for correlation analyses (Poldrack, 2007). This control ROI exhibited a lack of any activation either in 1- vs. 0-back or 2- vs. 1-back comparisons.

**Table 1:** Peak level activations in each cluster related to maintenance (1- vs. 0-back), manipulation (2- vs. 1-back), and maintenance with manipulation processes (2- vs. 0-back).

					MNI coordinates		
Cluster peak, hemisphere	Brodmann area (BA)	No. of voxels	*t*-value	*p*-value (FWE)	*x*	*y*	*z*
**1- vs. 0-back (maintenance)**
Middle frontal gyrus, L	6	74	6.069	0.001	−26	0	56
Middle frontal gyrus, R	6	18	5.840	0.001	44	4	56
Inferior parietal lobule, R	7	75	5.905	0.001	40	−40	40
Superior parietal lobule, L	7	19	5.888	0.001	−12	−70	54
Superior frontal gyrus, R	6	43	5.785	0.002	26	4	54
Inferior frontal gyrus, L	13	13	5.412	0.009	−30	22	8
Inferior frontal gyrus, L	44	10	5.256	0.016	−44	6	22
Medial frontal gyrus, L	8	14	5.366	0.010	−4	16	50
**2- vs. 1-back (manipulation)**
Insula, L	13	282	8.814	<0.0005	−30	24	2
Precuneus, R	7	255	8.320	<0.0005	10	−70	52
Cerebellum, L	−	251	8.134	<0.0005	−28	−66	−28
Cerebellum, R	−	65	6.590	<0.0005	28	−64	−30
Cerebellum, L	−	48	6.197	<0.0005	−12	−78	−26
Cerebellum, R	−	49	6.019	0.001	10	−74	−28
Middle frontal gyrus, R	9	754	7.856	<0.0005	38	40	32
Middle frontal gyrus, R	6	668	7.806	<0.0005	26	6	52
Middle frontal gyrus, L	6	451	7.189	<0.0005	−26	2	54
Middle frontal gyrus, L	46	207	6.660	<0.0005	−42	46	10
Middle frontal gyrus, L	9	284	6.428	<0.0005	−40	30	30
Inferior parietal lobule, R	40	984	7.768	<0.0005	48	−48	46
Inferior parietal lobule, L	40	1,083	7.111	<0.0005	−38	−44	40
Inferior frontal gyrus, R	13	308	7.304	<0.0005	32	28	2
Inferior frontal gyrus, L	6	69	6.448	<0.0005	−38	2	36
Medial frontal gyrus, L	8	535	7.280	<0.0005	−2	24	48
Superior frontal gyrus, L	6	42	6.149	<0.0005	−8	12	56
Superior frontal gyrus, L	10	10	5.540	0.005	−24	48	0
**2- vs. 0-back (maintenance with manipulation)**
Insula, L	13	3,379	11.349	<0.0005	−30	24	2
Inferior parietal lobule, R	40	1,963	10.340	<0.0005	38	−46	42
Inferior parietal lobule, L	40	2,058	9.579	<0.0005	−38	−44	40
Superior frontal gyrus, R	6	1,259	10.225	<0.0005	26	4	54
Superior frontal gyrus, L	6	1,120	8.471	<0.0005	−6	10	56
Cerebellum, L	−	334	8.980	<0.0005	−30	−68	−26
Cerebellum, R	−	192	8.141	<0.0005	28	−64	−30
Cerebellum, R	−	90	7.128	<0.0005	10	−76	−26
Cerebellum, L	−	44	6.310	<0.0005	−12	−76	−26
Inferior frontal gyrus, R	13	599	8.835	<0.0005	32	28	2
Middle frontal gyrus, R	9	717	8.310	<0.0005	38	38	30
Caudate, R	48	17	5.683	0.003	14	−2	14
Caudate, L	48	26	5.663	0.003	−16	0	16
Medial frontal gyrus, L	32	25	5.391	0.009	−6	28	34

*Presented p- and t-values were obtained with familywise error (FWE) correction at a voxel (peak) level. Regions were labeled using the Talairach Atlas labels. The *x*, *y*, and *z* Montreal Neurological Institute (MNI) coordinates were used for the left–right, anterior–posterior, and inferior–superior dimensions, respectively. In some structures, more peak activations were observed, but in each cluster, only one peak was indicated*.

Finally, the correlation analyses for these ROIs were conducted between mean brain activity extracted from 1- vs. 0-back and 2- vs. 1-back contrasts and obtained ATOT values. The MarsBaR toolbox was used to export brain activity^5^. To avoid inflated effects due to multiple comparisons, two-tailed Spearman’s rank correlations were applied with adjusted *p*-value cutoff. The cutoff was computed separately for the 1- vs. 0-back and 2- vs. 1-back comparisons, following Bonferroni correction, i.e., dividing a standard *p*-value of 0.05 by the number of performed correlations (Bland and Altman, 1995). As a result, for 1- vs. 0-back, a *p*-value of 0.006 (0.05/9 performed correlations) was used, whereas for the 2- vs. 1-back, a *p*-value of 0.003 (0.05/19 performed correlations) was used.

## Results

### Behavioral Indices of WM Task

**Figure 2:**
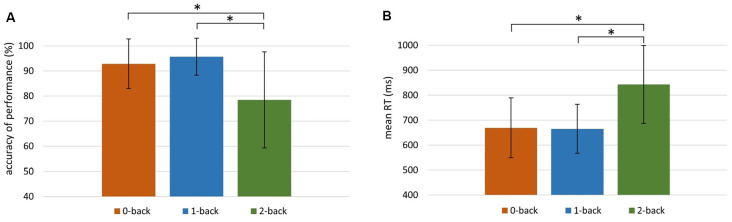
Behavioral indices of working memory (WM) efficiency in the 0-, 1-, and 2-back conditions. **(A)** Accuracy of performance (%) in 0-back: *M* = 92.83, *SD* = 9.92; 1-back: *M* = 95.66, *SD* = 7.33; 2-back: *M* = 78.41, *SD* = 19.18. **(B)** Mean reaction time (RT) in 0-back: *M* = 669 ms, *SD* = 121 ms; 1-back: *M* = 665 ms, *SD* = 98 ms; 2-back: *M* = 843 ms, *SD* = 156 ms. Significant differences (*p* < 0.001) are indicated with an asterisk.

The behavioral data obtained in each *n*-back condition are presented in Figure 2. It displays the accuracy of performance (Figure 2A) and the mean RT (Figure 2B) in 0-, 1-, and 2-back conditions.

The Friedman test revealed significant differences between the accuracy of performance in these three conditions ($\chi_{\left(41,2\right)}^{2}$ = 35.30, *p* < 0.001). The accuracy in the 2-back condition was significantly lower than in the other two conditions (*p* < 0.001; Wilcoxon tests). There were also significant differences between mean RTs in the 0-, 1-, and 2-back conditions ($\chi_{\left(41,2\right)}^{2}$ = 48.20, *p* < 0.001; Friedman test). The mean RT was higher in the 2-back condition than in the 0- and 1-back conditions (*p* < 0.001; Wilcoxon tests).

### Psychophysical Indices of TIP

In the present study, the median ATOT value was 89 ms. This is consistent with the results of our previous studies where ATOT values in elderly participants were between 60 and 100 ms (Szymaszek et al., 2009; Szelag et al., 2018).

### Correlations Between Behavioral Indices of WM and ATOT Values

**Figure 3:**
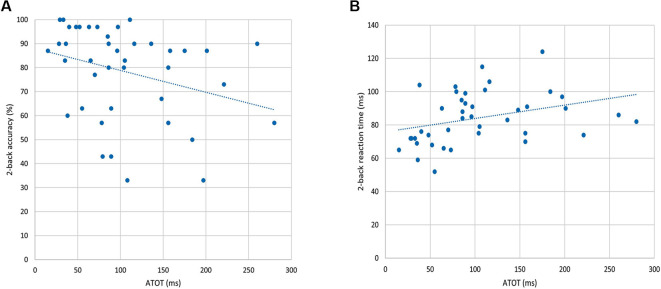
Correlations between behavioral indices from the 2-back condition and auditory temporal-order threshold (ATOT) values: **(A)** accuracy of performance and **(B)** mean RT.

To investigate the “WM–TIP” relationship, partial correlations between *n*-back behavioral indices (in 0-, 1-, and 2-back conditions) and ATOT were conducted, controlling for age. Only in the 2-back condition did the results show significant correlations between ATOT values and accuracy (*rho* = −0.37, *p* < 0.05; Figure 3A), as well as between ATOTs and mean RTs (*rho* = 0.47, *p* < 0.01; Figure 3B). This indicates that better performance on the WM task (reflected in higher accuracy and shorter mean RT) was accompanied by more efficient TIP (lower ATOT values). In 0- and 1-back conditions, these correlations were nonsignificant.

### fMRI Results of the *n*-Back Task

**Figure 4:**
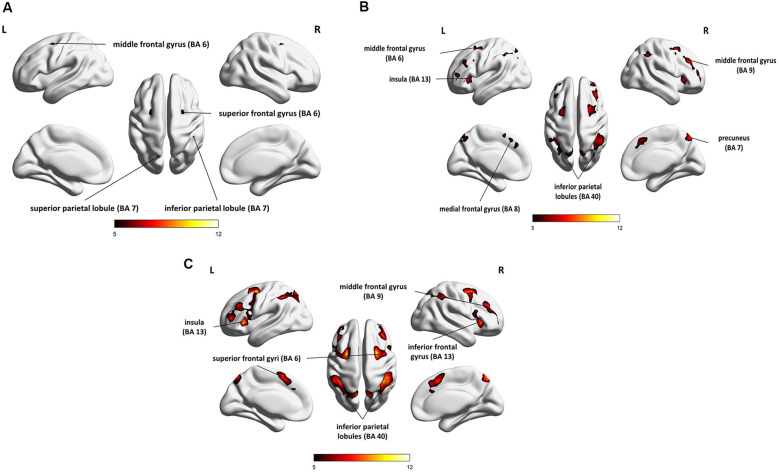
Whole-brain statistical parametric maps representing brain activation specific to: **(A)** maintenance (1- vs. 0-back), **(B)** manipulation (2- vs. 1-back), and **(C)** maintenance with manipulation (2- vs. 0-back). A voxel-level familywise error (FWE) correction was applied (*p* < 0.05) with a cluster extent threshold of 10. L, left hemisphere; R, right hemisphere.

The results of the group analysis are presented in Table 1 and Figures 4A–C.

Direct comparison of brain activity in the 1- vs. 0-back condition (Figure 4A) showed regions engaged mostly in WM maintenance processes (for a more detailed discussion of maintenance/manipulation processes, see “Introduction” section). The comparison revealed activations in bilateral MFG (BA 6), right IPL (BA 7), left superior parietal lobule (SPL; BA 7), right superior frontal gyrus (SFG; BA 6), and left inferior frontal gyrus (IFG; BA 13 and 44), indicating the engagement of the vlPFC, as well as the left medial frontal gyrus (BA 8).

On the other hand, the areas involved mainly in manipulation processes were identified by contrasting the 2- and 1-back conditions (Figure 4B). This comparison showed, in general, stronger and wider activations than those observed in the 1- vs. 0-back comparison (maintenance). These activations comprised the left insula (BA 13), right precuneus (BA 7), bilateral cerebellum, and MFG (BA 6, 9, and 46), indicating the involvement of the dlPFC, bilateral IPL (BA 40), and IFG (BA 6 and 13), as well as the left medial frontal gyrus (BA 8) and left SFG (BA 6 and 10).

The 2- vs. 0-back comparison (reflecting maintenance with manipulation; Figure 4C) also revealed a widespread network of fronto-parieto-cerebellar activations, comprising the left insula (BA 13), bilateral IPL (BA 40), SFG (BA 6), and cerebellum, as well as the right IFG (BA 13) and MFG (BA 9), bilateral caudate (BA 48), and left medial frontal gyrus (BA 32).

### Correlations Between Brain Activity in WM and the Efficiency of TIP

**Figure 5:**
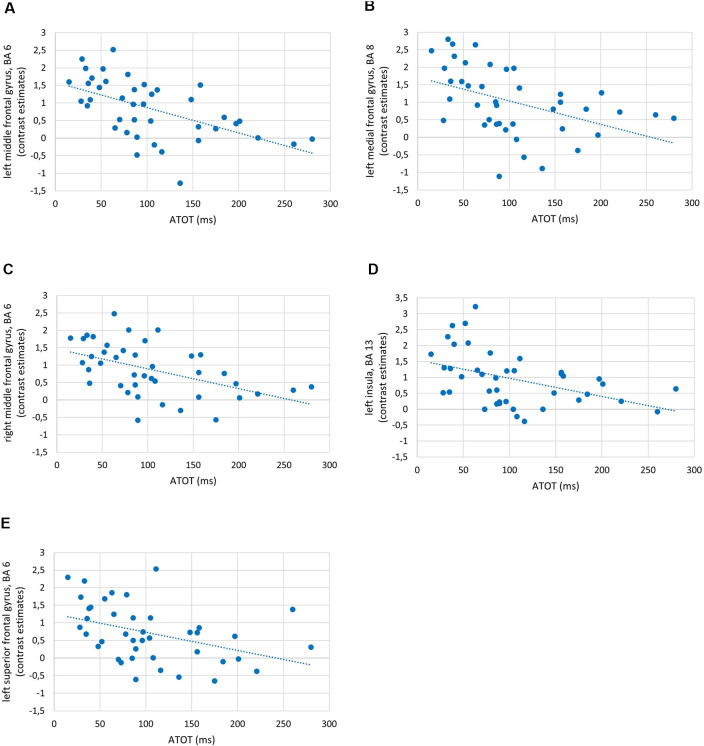
Examples of correlations between temporal-order threshold (ATOT) values and brain activity in regions of interest (ROIs) with peak level activation in **(A)** left middle frontal gyrus (MFG), BA 6; **(B)** left medial frontal gyrus, BA 8; **(C)** right MFG, BA 6; **(D)** left insula, BA 13; and **(E)** left superior frontal gyrus (SFG), BA 6.

To investigate the relationships between the efficiency of TIP and brain activity in WM processes (maintenance/manipulation), we calculated correlations between ATOT values and mean activation extracted from ROIs (the ROI selection is described in “Statistical Analyses” section). The results revealed moderate negative correlations with some frontal brain regions, as well as the insula, engaged in manipulation processes (2- vs. 1-back). Correlations were found in the bilateral MFG (BA 6, 9, and 46), left medial frontal gyrus (BA 8), left insula (BA 13), and left SFG (BA 6). This indicates that more efficient TIP (reflected in lower ATOTs) was related to stronger activations only in the above regions during the WM manipulation process (Figure 5). In contrast, no significant correlations in regions related to maintenance (1- vs. 0-back) were observed. The outcomes of the correlation analyses are presented in Table 2.

**Table 2:** Correlations (*rho* and *p*-values) between temporal information processing (TIP) and brain activity in regions of interest (ROIs) reflecting working memory (WM) manipulation (2- vs. 1-back) and maintenance (1- vs. 0-back).

Cluster peak, hemisphere	Brodmann area (BA)	No. of voxels	MNI coordinates			*Rho*	*p*
			*x*	*y*	*z*		
*2- vs. 1-back (manipulation)*
**Middle frontal gyrus, L**	**6**	**451**	**−26**	**2**	**54**	**−0.616**	**<0.0005**
**Middle frontal gyrus, L**	**46**	**207**	**−42**	**46**	**10**	**−0.583**	**<0.0005**
**Medial frontal gyrus, L**	**8**	**535**	**−2**	**24**	**48**	**−0.579**	**<0.0005**
**Middle frontal gyrus, R**	**6**	**668**	**26**	**6**	**52**	**−0.561**	**<0.0005**
**Middle frontal gyrus, R**	**9**	**754**	**38**	**40**	**32**	**−0.550**	**<0.0005**
**Insula, L**	**13**	**282**	**−30**	**24**	**2**	**−0.529**	**<0.0005**
**Superior frontal gyrus, L**	**6**	**42**	**−8**	**12**	**56**	**−0.520**	**0.001**
**Middle frontal gyrus, L**	**9**	**284**	**−40**	**30**	**30**	**−0.457**	**0.003**
Inferior frontal gyrus, R	13	308	32	28	2	−0.450	0.004
Superior frontal gyrus, L	10	10	−24	48	0	−0.441	0.004
Inferior parietal lobule, L	40	1,083	−38	−44	40	−0.426	0.006
Inferior parietal lobule, R	40	984	48	−48	46	−0.372	0.018
Precuneus, R	7	255	10	−70	52	−0.359	0.023
Cerebellum, R	–	49	10	−74	−28	−0.303	0.057
Cerebellum, R	–	65	28	−64	−30	−0.279	0.081
Inferior frontal gyrus, L	6	69	−38	2	36	−0.261	0.103
Cerebellum, L	–	48	−12	−78	−26	−0.256	0.111
Cerebellum, L	–	251	−28	−66	−28	−0.245	0.127
Control ROI	18	4,120	10	−80	20	−0.141	0.386
*1- vs. 0-back (maintenance)*
Middle frontal gyrus, L	6	74	−26	0	56	−0.203	0.209
Inferior parietal lobule, R	7	75	40	−40	40	0.027	0.868
Superior parietal lobule, L	7	19	−12	−70	54	−0.047	0.772
Middle frontal gyrus, R	6	18	44	4	56	−0.167	0.302
Superior frontal gyrus, R	6	43	26	4	54	0.027	0.871
Inferior frontal gyrus, L	13	13	−30	22	8	−0.281	0.079
Medial frontal gyrus, L	8	14	−4	16	50	−0.131	0.421
Inferior frontal gyrus, L	44	10	−44	6	22	0.156	0.335
Control ROI	18	4,120	10	−80	20	−0.277	0.084

*The p-value cutoff with Bonferroni correction for 2- vs. 1-back was 0.003, and for 1- vs. 0-back, it was 0.006 (for further explanation, see “*Materials andMethods” section*). Significant correlations are in bold*.

## Discussion

### Summary of Results

Using the auditory verbal *n*-back task, we found evidence in elderly people of the activity of a WM brain network comprising mainly the prefrontal cortex (MFG, SFG, IFG), IPL, and SPL, as well as the insula, precuneus, and cerebellum. The relevant result from the present study was the indication of similar brain networks that differ in the range and intensity of activity for the two WM processes, i.e., maintenance (reflected in 1- vs. 0-back comparisons) and manipulation (2- vs. 1-back). For maintenance, the activity was lower and more focused in comparison to that found for manipulation, which was characterized by higher and more spread-out activity (Table 1). These effects corresponded to behavioral data that indicated the lower accuracy of performance and longer RT in the 2-back condition than in the 1- or 0-back conditions, confirming that the 2-back condition (based mainly on manipulation) is more difficult than two other conditions studied here (see Figure 2).

Our results also provided a new, previously unstudied, insight into the “WM–TIP” relation evidenced in both behavioral data and brain activity. We observed moderate significant correlations between psychophysical indices of TIP (ATOT values) and behavioral 2-back performance (accuracy and RTs; Figure 3), as well as brain activity during manipulation processes (2- vs. 1-back comparisons; Table 2). The better temporal resolution (i.e., lower ATOT) was accompanied by better performance on the 2-back task and stronger activations in regions engaged in manipulation. In contrast, these correlations proved nonsignificant in 1- and 0-back conditions and in maintenance processes, indicating a weaker contribution of temporal resolution to maintenance. Such correlations allowed the identification of some brain areas within the WM network, which are also strongly related to temporal resolution.

### Brain Network Supporting WM

#### Maintenance

The brain network engaged in maintenance reported here (Figure 4A) is similar to that reported in previous studies (Tsukiura et al., 2001; Ragland et al., 2002; Crottaz-Herbette et al., 2004; Narayanan et al., 2005) and comprised fronto-parietal regions, including the bilateral MFG (BA 6), right SFG (BA 6), left IFG (BA 13, 44), and left medial frontal gyrus, as well as the right IPL (BA 7) and left SPL (BA 7). It should be noted that the network reported in the above studies was independent of the subjects’ age (remaining similar across the lifespan from 18 to 53 years), target modality (visual, auditory), and the procedure used.

A similar brain network as evidenced in our study for maintenance, comprising the left hemispheric premotor cortex (BA 6), SFG (BA 8), and precentral gyrus (BA 44), was previously reported using different procedures. For example, Narayanan et al. (2005) used an item recognition task and found that increasing memory load (task difficulty) was related to increasing activity in the dlPFC and vlPFC. Moreover, the parietal activations in the IPL and SPL (BA 7) evidenced by us indicate the contribution of information storage, which has been frequently observed in the literature (Awh et al., 1996; Jonides et al., 1998). Our results also indicate the contribution of the vlPFC (reflected in BA 44 activations) to maintenance, which is congruent with the findings of Owen (1997) and D’Esposito et al. (1998).

On the other hand, some reports also suggest activity in the dlPFC, specifically in the right MFG, during maintenance (BA 9, 46; Tsukiura et al., 2001), which was not observed in our study. This disagreement between our data and those obtained by Tsukiura et al. (2001) may be due to the more complicated procedure used, causing more complex maintenance processes with an associated delay in the subjects’ reaction. It is possible that the complex task used by Tsukiura et al. (2001) may activate a larger network of structures than the relatively simple task used in our study.

#### Manipulation

Although in our study the brain network engaged in maintenance and manipulation processes was nearly the same, the network involved in manipulation was characterized by higher and more spread-out activity (Figures 4A,B). This probably reflects the more complex processes required for manipulation, which involves the permanent reorganization and updating of analyzed material.

In line with previous studies, the manipulation-specific regions in our study were the bilateral MFG (BA 6, 9, 46), IFG (BA 6, 13) and IPL (BA 40), left SFG (BA 6, 10) and insula (BA 13), and also the bilateral cerebellum. These results support the broad engagement of the core WM system in the fronto-parietal network during manipulation (Tsukiura et al., 2001; Ragland et al., 2002; Veltman et al., 2003; Schmidt et al., 2009; Barbey et al., 2013). In agreement with the traditional functional division between maintenance and manipulation, our results suggest that activity in the dlPFC (BA 9 and 46), but not in vlPFC (BA 44, 45, 47), is involved in manipulation.

It is also important to emphasize the involvement of the cerebellum in the WM network in our study. Cerebellar activations during WM tasks have also been reported in previous studies (Luis et al., 2015); therefore, our results support the involvement of the fronto-parieto-cerebellar network in WM. Interestingly, the contribution of motor system network was indicated in both verbal and nonverbal WM performance (Liao et al., 2014). Cooper et al. (2012) suggested that cerebellum plays an integrative role in processing of motor information and pointed out importance of these integrative processes also in executive functions and other cognitive tasks. Cerebellum involvement was observed not only in WM but also in various TIP tasks (Ben-Yehudah et al., 2007). The activations in the cerebellar cortex in the elderly reported in our study may be due to the contribution of compensatory mechanisms supporting more complex processing during manipulation, which may be more difficult for elderly subjects, taking into account age-related decline.

To sum up, our results are generally in line with previous reports that used the *n*-back task in elderly subjects, supporting the engagement of the broad fronto-parieto-cerebellar network in WM processes (Owen et al., 2005; Luis et al., 2015).

#### Functional Hemispheric Asymmetry in Verbal WM in the Aging Brain

According to the literature (Owen et al., 2005), the type of material used in the *n*-back task in the present study (syllables) may primarily activate left hemispheric regions. The language-dominant hemisphere usually exhibits stronger involvement in memory processing of verbal material (Bradshaw and Nettleton, 1981; Gazzaniga, 2000; Weber et al., 2007; Hellige, 2008). It may be supported by electrical neuroimaging analyses of auditory evoked potentials recorded while the participants completed the TOJ task (Bernasconi et al., 2010, 2011). They showed the engagement of the left, but not right, posterior perisylvian regions activity as a predictor of accurate behavioral performance of auditory TOJ task and early encoding phases of paired stimuli critical for the perception of their order. However, in our study, despite the syllable processing required (i.e., typical verbal material), there was no clear lateralization pattern in either maintenance or manipulation processes—we observed left, bilateral, and right activations of brain structures (Figure 4). As stated above, the observed left hemispheric activity is consistent with previous findings (Owen et al., 2005) and confirms that lateralization is determined by the type of presented material. As we also found bilateral and right hemispheric activations in verbal stimuli processing, two possible explanatory mechanisms may be proposed for the observed hemispheric asymmetry pattern.

First, despite the general left hemispheric dominance in language, there is also much evidence in the literature regarding the contribution of the right hemisphere (Hellige, 2001, 2008). This lateralization pattern may result from the involvement of global holistic processing in language processing. In our study, it may support the phonological sequential analysis of presented syllables, which may be also processed as whole acoustic patterns without the need for linguistic coding (Zatorre and Samson, 1991; Hellige, 2001; Lindell, 2006).

Second, the observed bilateral activations may be associated with age-related reduced hemispheric asymmetry. Previous research revealed that elderly adults demonstrate reduced material-relevant activity and engagement of additional, nonspecific brain areas in comparison to young adults performing the same task (Cabeza, 2002; Mattay et al., 2002). This extra activity is interpreted as a neurocognitive compensatory mechanism underlying the dedifferentiation hypothesis of hemispheric asymmetry (Dennis and Cabeza, 2008). Accordingly, brain areas become less specialized with increasing age, leading to decreased activity in task-relevant regions, accompanied by increased activity in less specialized brain areas. Therefore, bilateral and right hemispheric activations in our study could result from an interplay of such compensatory mechanisms. Thus, to meet the demands of the maintenance and manipulation processes, additional engagement of cross-hemispheric counterparts is observed in the elderly.

### The “WM–TIP” Relationship

#### The Role of TIP in WM Processes

As mentioned in “Introduction” section, previous studies have suggested that TIP plays an important role in numerous cognitive functions, such as language, attention, learning, executive processes, and WM (Pöppel, 1997; Poeppel, 2003; Szymaszek et al., 2009; Ulbrich et al., 2009; Nowak et al., 2016). Because of the specific temporal dynamics of these functions, it can be assumed that they are rooted in a defined time template creating the neural frame for our mental activity. Despite pronounced individual differences, experimental data have indicated that proper temporal ordering is controlled by a hypothetical internal clock (pacemaker counter or oscillator device), likely operating at a high oscillation rate, within a window of some tens of milliseconds (Treisman, 1963; Treisman et al., 1990; Pöppel, 1997; VanRullen and Koch, 2003; Rammsayer and Brandler, 2007; Troche and Rammsayer, 2009; Binder, 2015).

The temporal dynamics of WM are emphasized in the classic Scalar Expectancy Theory (see articles cited in “Introduction” section), which assumes that TIP is attributable to the operation of WM. Despite these facts, studies on the neural basis of WM have often ignored the underlying time frame in which the specific WM processes are embedded.

WM and TIP are fundamental properties of mental activity and commonly believed to interact with each other. The influences of many other factors are an important aspect of this interaction. Our paradigm employed context-specific processes, i.e., auditory perception of exposed syllables, phonological processing, rehearsal resources, and executive properties (comparison, decision-making, motor reaction, or suppression of some reactions). In the subject sample studied here, we confirmed the existence of individual differences in TIP and WM efficiency, evidenced in behavioral data (Figure 3). The question is: *what mechanisms coexist and cooperate with such multifactorial WM resources?*

#### Clock Functions Differentiate WM Processes

The novel value of the present study is the observation of a large difference, never reported before, between two different WM processes (maintenance/manipulation) in relation to their TIP properties. Both the behavioral data (Figure 3) and brain activity registered in the scanner (Figure 5) indicated a strong contribution of TIP to manipulation, but not to maintenance (Table 2). *Why are temporal dynamics incorporated differently in these two processes?*

In the 1-back condition, involving mostly maintenance (storage and rehearsal), the incoming information is collected by integrating information over intervals of ~2-s duration, referring to the last syllable followed by the button press (or the suppression thereof), while minimizing the role of updating permanent resources. In the 0-back condition, such integration intervals were much longer because the predefined target syllable (here WO) remained unchanged during the whole trial.

In contrast, the necessity of manipulation in the 2-back condition (permanent reorganizing and updating) incorporated the strong temporal dynamics in a millisecond frame during relatively long intervals. Such integration intervals were not only longer than those in the 1-back condition but also required more advanced phonological analysis in order to distinguish between phonemes in processed consonant–vowel syllables. In such processing, skilled millisecond timing (proper temporal resolution) is crucial for decoding the initial stop consonants which, in many languages, are limited in time up to ~40 ms. In such short intervals, rapid formant transitions characteristic to the initial consonants must be decoded. During this phonological processing, high temporal resolution is necessary for the proper identification and correct ordering of consonants and vowels in the presented syllables (Poeppel, 2003; Szelag et al., 2014). Additionally, in the 2-back condition, the information was collected and analyzed by integrating information in real time. This relies on continuous online updating and comparison with the syllable before–last, accompanied by a tentative suppression of the last syllable. Such manipulation transcended the storage required for maintenance by the dynamic processing of information with a precision of some tens of milliseconds. As a consequence, manipulation caused the 2-back condition to be more difficult than the 1- or 0-back ones (Figures 2, 3).

To understand better the “WM-TIP” relationships, it should be clarified whether they may be also evidenced using other temporal processing tasks. The previous studies, including studies conducted in our laboratory, indicated the heterogeneity of temporal ordering ability measured with various TOJ tasks. In these studies, the obtained absolute threshold values were strongly stimulus-dependent, procedure-related, and influenced by perceptual strategies used by participants (Szymaszek et al., 2009; Ulbrich et al., 2009; Fostick and Babkoff, 2017; Szelag et al., 2018). Furthermore, these threshold values depended on peripheral sensory mechanisms, corresponding to shorter transduction time for auditory than visual stimuli at the level of receptive cells in each modality system (Kanabus et al., 2002).

The question is whether the relationship reported here may be also observed using other tasks addressing millisecond TIP. Although the present experiment cannot answer this question directly, we are of the opinion that the binaural presentation of two tones differing in pitch applied here may reflect properties of the hypothetical internal clock (see the Scalar Expectancy Theory, Matthews and Meck, 2016). Further experimental studies are necessary to clarify these issues.

These considerations lead to another question that could be answered in our recently running research project, whether the “WM-TIP” relationships are characteristic selectively for millisecond TIP or also for other temporal levels, namely, several hundred millisecond or multisecond domains. There is some evidence that the interval timing (addressed usually multisecond level) and WM can originate from the same oscillatory brain activity and may share common cognitive properties, such as attentional or executive resources (for the recent review see Gu et al., 2015). These common properties were reviewed from behavioral, anatomical, pharmacological, and neuropsychological perspectives.

On this background, the novel value of our study is the indication of some dissociation in WM processes, indicating the temporal constraints of manipulation but rather not of maintenance processes. Accordingly, our finding gives a new light on relationships between WM and TIP, which are critical components of cognition.

#### Correlations Between ATOT and Behavioral and Neuroimaging WM Indices

In terms of individual resources in TIP, persons characterized by better temporal resolution (lower ATOT in our study) could follow the updating in real time more accurately and with shorter RT, as evidenced in Figure 3. It is important to stress that the above processing was important for the 2-back condition, which mostly required manipulation, but not for the 1- and 0-back conditions, which mostly involved maintenance (compare above).

As the behavioral WM indices corresponded strongly to brain activity, we observed in parallel significant moderate negative correlations between temporal resolution evidenced by psychophysical TIP indices (ATOT values) and brain activity in 2- vs. 1-back comparisons (Figure 5). In other words, for manipulation, better temporal resolution (lower ATOT) was accompanied by higher activity (corresponding to more neural resources involved). Such activity comprised (Table 2) mostly the dlPFC (specifically, MFG: bilateral BA 9 and left BA 46), accompanied by the bilateral MFG (BA 6), left medial frontal gyrus (BA 8), SFG (BA 6), and insula (BA 13). In contrast, less skilled TIP (lower temporal resolution) was related to less efficient manipulation, evidenced in lower activity in these areas, accompanied by lower accuracy of performance with longer RTs (Figures 3, 5).

In contrast, in maintenance (1- vs. 0-back comparisons), such correlations proved nonsignificant (Table 2) probably because of a weak contribution of temporal dynamics to storage processes.

To explain correlations between TIP and brain activity in WM, one should refer to general age trends in functional brain activation during WM performance. As stated in “Introduction” section, both WM and TIP decline in advanced age. The pattern of activations in aging brain is not easily interpretable, as some studies report underactivation of task-relevant brain areas, whereas others find overactivation relative to younger adults (e.g., Reuter-Lorenz, 2002; for the recent overview see Nagel and Lindenberger, 2015). Accordingly, underactivation is interpreted as a sign of structural, neuromodulatory, and hemodynamic response declines. Overactivation, on the other hand, is reported as a form of larger activation in elderly. The reduction in processing efficiency in elderly may lead to compensatory reactions, which occur in a response to deficient processing. The activation of additional regions may function as an aid to preserve WM (and probably other cognitive functions) because of age-related losses (Park and Reuter-Lorenz, 2009). Considering these evidence, we are of the opinion that TIP may support WM processes, especially during manipulation, which involves more complex resources than the maintenance. The stronger activity in manipulation may reflect the contribution of more efficient TIP into WM to support such age-related WM losses. Further studies are needed to explain these issues in detail.

#### Brain Activity Overlaps in WM and TIP

Support for the “WM–TIP” relation in manipulation reported here can be found in studies on the neuroanatomy of TIP. Considering the broad neural network activated in WM manipulation identified in this study (Table 1), we found selected areas within this WM network that are strongly related to the effectiveness of temporal ordering (Figure 5, Table 2). These areas do not only concern WM function but are thought to also reflect the cognitive demands of the TIP network reported in the literature on the neuroanatomy of TIP. Based on the meta-analysis provided by Lewis and Miall (2003), supported by subsequent reports (Lewis and Miall, 2006; Lewandowska et al., 2010; Kotz and Schwartze, 2011), PFC (both dorso- and ventrolateral), pre-SMA, frontal pole, and parietal network activity were often reported in TIP. Referring to the diverse timing representation model proposed by Grondin (2010) and Merchant et al. (2013), assuming a partially shared timing mechanism, the dlPFC activity may reflect the contribution of nontemporal resources such as WM and comparison functions during TIP tasks (Rao et al., 2001; Livesey et al., 2007), irrespective of stimulus modality (Pastor et al., 2006) or task difficulty (Livesey et al., 2007). On the other hand, insular activation has also been observed in literature studies on paradigms involving auditory TIP, phonological processing, and sound detection (Craig, 2002, 2009).

To sum up, our results are in agreement with the partially shared timing theoretical model that assumes that TIP depends on the interaction of multiple areas, including main core TIP areas and context-dependent areas. The interaction between these two types of areas controls the specific performance of the task (Merchant et al., 2013). On the basis of our neuroimaging data, it may be concluded that the neural network responsible for the updating during dynamic manipulation of resources in WM is also sensitive to temporal ordering in the millisecond time window.

The data reported here may have not only the theoretical relevance but also the practical importance. They support the thesis that accurate WM manipulation processes require accurate TIP resources. Thus, the application of specific training in TIP may result in a transfer of improvement from time domain to WM domain *via* improvement of WM manipulation, but not maintenance, resources (Szelag et al., 2015a,b; Szymaszek et al., 2018). It would provide a new light on neurorehabilitation of subjects suffering from declined WM functions.

Finally, it would be interesting for future studies to investigate whether in traditional item-recognition WM tasks (e.g., the Sternberg task), often classified as storage tasks (Veltman et al., 2003), correlations between the efficiency of TIP and WM could be also observed. As some previous studies postulated the involvement of the dlPFC mainly during manipulation processes, in which the pure storage of information is less engaged (Marvel and Desmond, 2012), one might anticipate a lack of significant correlations between the efficiency of WM and TIP in these item-recognition tasks.

### Final Remarks

The relationships observed here contribute to the existing literature some important new data on the neural network controlling WM, supporting the differentiation between processes engaged in the *n*-back task. The most important result of this study is the indication of the divergent involvement of TIP in maintenance and manipulation WM processes. We identified for the first time the contribution of temporal properties to brain activity—but only in manipulation processes, which require the continuous reorganization and updating of incoming information. It appears that this relation cannot work for processes that rely more heavily on storage maintenance.

## Data Availability Statement

The datasets generated for this study are available on request to the corresponding author.

## Ethics Statement

The studies involving human participants were reviewed and approved by Ethical Commission at the University of Social Sciences and Humanities (permission no. 1/2017, registered as 2 /I/ 16-17). The patients/participants provided their written informed consent to participate in this study.

## Author Contributions

KJ and MP: subject recruitment, acquisition, analysis, and interpretation of data. HB and AS: interpretation of data and manuscript writing. AM and MW: analysis and interpretation of fMRI data. ES: conceptualization and study design, analysis and interpretation of data, manuscript writing, and responsibility for the final version of manuscript.

## Conflict of Interest

The authors declare that the research was conducted in the absence of any commercial or financial relationships that could be construed as a potential conflict of interest.

## Footnotes

^1^www.neurobs.com

^2^http://www.fil.ion.ucl.ac.uk/spm/software/spm12/

^3^https://www.nitrc.org/projects/artifact_detect

^4^http://www.alivelearn.net/xjview/

^5^http://marsbar.sourceforge.net/

## Abbreviations

ACC: anterior cingulate cortex
aPFC: anterior prefrontal cortex
ATOT: auditory temporal-order threshold
BG: basal ganglia
dlPFC: dorsolateral prefrontal cortex
IFG: inferior frontal gyrus
IPL: inferior parietal lobule
ISI: interstimulus interval
MFG: middle frontal gyrus
ROI: region of interest
RT: reaction time
SFG: superior frontal gyrus
SMA: supplementary motor area
SPL: superior parietal lobule
TIP: temporal information processing
TOJ: temporal-order judgment
vlPFC: ventrolateral prefrontal cortex
WM: working memory.
